# Effects of postponing nitrogen topdressing on starch structural properties of superior and inferior grains in hybrid *indica* rice cultivars with different taste values

**DOI:** 10.3389/fpls.2023.1251505

**Published:** 2023-10-10

**Authors:** Xiaojuan Yuan, Yongheng Luo, Yonggang Yang, Kairui Chen, Yanfang Wen, Yinghan Luo, Bo Li, Yangming Ma, Changchun Guo, Zongkui Chen, Zhiyuan Yang, Yongjian Sun, Jun Ma

**Affiliations:** ^1^ Crop Ecophysiology and Cultivation Key Laboratory of Sichuan Province, Sichuan Agricultural University, Chengdu, China; ^2^ Key Laboratory of Southwest Rice Biology and Genetic Breeding, Ministry of Agriculture and Rural Affairs, Rice and Sorghum Research Institute, Sichuan Academy of Agricultural Sciences, Deyang, China; ^3^ State Key Laboratory of Crop Gene Exploration and Utilization in Southwest China, Sichuan Agricultural University, Chengdu, Sichuan, China

**Keywords:** *indica* rice, postponing nitrogen topdressing, starch structure, superior and inferior grains, eating quality

## Abstract

**Introduction:**

Nitrogen (N) fertilizer management, especially postponing N topdressing can affect rice eating quality by regulating starch quality of superior and inferior grains, but the details are unclear. This study aimed to evaluate the effects of N topdressing on starch structure and properties of superior and inferior grains in hybrid *indica* rice with different tastes and to clarify the relationship between starch structure, properties, and taste quality.

**Methods:**

Two hybrid *indica* rice varieties, namely the low-taste Fyou 498 and high-taste Shuangyou 573, were used as experimental materials. Based on 150 kg·N hm^-2^, three N fertilizer treatments were established: zero N (N_0_), local farmer practice (basal fertilizer: tillering fertilizer: panicle fertilizer=7:3:0) (N_1_), postponing N topdressing (basal fertilizer: tillering fertilizer: panicle fertilizer=3:1:6) (N_2_).

**Results:**

The starch granules of superior grains were more complete, and the decrease in small granules content and the stability of starch crystals were a certain extent less than those of inferior grains. Compared with N_1_, under N_2_, low-taste and high-taste varieties large starch granules content were significantly reduced by 6.89%, 0.74% in superior grains and 4.26%, 2.71% in inferior grains, the (B2 + B3) chains was significantly reduced by 1.61%, 0.98% in superior grains, and 1.18%, 0.97% in inferior grains, both reduced the relative crystallinity and 1045/1022 cm^-1^, thereby decreasing the stability of the starch crystalline region and the orderliness of starch granules. N_2_ treatment reduced the ΔHgel of two varieties. These changes ultimately contributed to the enhancement of the taste values in superior and inferior grains in both varieties, especially the inferior grains. Correlation analysis showed that the average starch volume diameter (D[4,3]) and relative crystallinity were significantly positively correlated with the taste value of superior and inferior sgrains, suggesting their potential use as an evaluation index for the simultaneous enhancement of the taste value of rice with superior and inferior grains.

**Discussion:**

Based on 150 kg·N hm^-2^, postponing N topdressing (basal fertilizer: tillering fertilizer: panicle fertilizer=3:1:6) promotes the enhancement of the overall taste value and provides theoretical information for the production of rice with high quality

## 1 Introduction

Rice is the main food crop and staple food in Asian countries (Chen et al., 2023). As the quality of life improves, the demand for rice with excellent taste has been increasing steadily (Jiang et al., 2022a). Rice quality is influenced by numerous genetic factors, and the underlying genetic mechanisms are complex. Starch constitutes the component of rice, accounting for approximately 90% of the dry matter weight of milled rice. This indicates that starch plays a crucial role in rice quality traits (Chen et al., 2021; Xiong et al., 2022a). Among the factors affecting starch quality, amylose is considered the most significant and is closely associated with rice cooking, texture, and other characteristics (Wani et al., 2012; Zhou et al., 2020). In general, a higher proportion of amylose contributes to increasing rice hardness, thereby diminishing its palatability (Zhou et al., 2015; Li and Gong, 2020; Zhang et al., 2023). In addition, starch properties are also regulated by amylopectin (Zhu et al., 2021). Such as rice with a higher amylopectin average chain length, and a higher proportion of long-chain (B3) amylopectin, increases the relative crystallinity and gelatinization temperature, which makes cooked rice harder. (Cao et al., 2017; Hu et al., 2020; Zhou et al., 2020). Similarly, rice with a higher proportion of short-chain (A and B1 chain) amylopectin, which is stickier after cooking (Li and Gilbert, 2018).

The quality of the rice varies depending on its position on the panicle. In general, superior grains that grow in the middle and upper parts of the panicle show early flowering, good grain filling, and high eating quality, whereas inferior grains that grow in the lower part of the panicle show late flowering, slow rate filling, poor filling, and low taste quality (Jiang et al., 2022b). The quality of superior and inferior grains directly affects the quality of rice. In particular, inferior grains are the key to limiting the overall quality improvement of rice. Therefore, it is of great significance to explore the difference in starch fine structure between superior and inferior grains for improving the quality of the whole panicle of rice. Fewer studies have been conducted on the effects of variations in starch structure and physicochemical properties of superior and inferior grains on eating quality. Jiang et al. (2022a) reported that compared with superior grains, inferior grains have a high content of A chains and low B2 and B3 chain contents of amylopectin, resulting in lower relative crystallinity and higher starch surface order. This increases starch gelatinization enthalpy and retrogradation enthalpy, reduces starch swelling potential and solubility, deteriorates starch gelatinization characteristics, reduces the taste value of inferior grains, and hardens the texture of rice. Jiang et al. (2022b) showed that the lower eating quality of inferior grains can be attributed to smaller starch granules and reduced crystallinity and order degree. These factors lead to a less stable starch crystal structure, resulting in increased breakdown value, decreased setback value, and compromised characteristic parameters of the rice starch rapid viscosity analysis (RVA) spectrum. However, Zhu et al. (2021) contradicted this finding by showing that the cooking and eating quality of inferior grains was higher than that of superior grains. They attributed this difference to the lack of abundant amylose, a high proportion of amylopectin A chains, and a low proportion of B3 chains in inferior grains. The starch granule size and relative crystallinity of the inferior grains decreased, and the crystal structure of starch was destroyed, thereby reducing the gelatinization enthalpy and gelatinization temperature, increasing stickiness, and reducing the hardness of the inferior grains.

Nitrogen (N) fertilizer is a key element for rice yield and quality (Dou et al., 2017). The application amount and management mode of N fertilizer affect the structure and properties of starch, which in turn affects taste quality. Excessive N fertilization application increases the relative crystallinity of starch but reduces rice quality, especially its palatability (Zhu et al., 2017). Conversely, balanced N application enhances starch granule traits, increases the proportion of short (A chain, B1 chain) amylopectin, and optimizes starch thermal properties, swelling properties, and RVA profile characteristics. These factors contribute to the improvement in rice taste value (Yang et al., 2022a).

Low N panicle fertilizer application rate reduced the α-1,6 linkage content and amylopectin short chains content and disrupted the long chain double helix structure of amylopectin, increased breakdown, and decreased the setback, thereby improving rice cooking and eating quality (Xiong et al., 2023). However, high N panicle fertilizer application rate increased the surface order of starch and the stability of the crystalline region. As a result, the swelling and gelatinization of starch granules are hindered, thereby reducing the taste value of rice (Jiang et al., 2023). Based on the total nitrogen application rate of 270 kg N hm^-2^, postponing N topdressing (basal fertilizer: tillering fertilizer: panicle fertilizer=4:2:4) optimizes the starch granule properties of superior and inferior grains, reduces the values of 1045/1022 cm^-1^ and relative crystallinity, increases gel consistency, and improves eating quality (Jiang et al., 2022b). Zhou et al. (2022) found that based on the total nitrogen application rate of 270 kg N hm^-2^, the postponing of nitrogen fertilizer (basal fertilizer: tillering fertilizer: panicle fertilizer=4:2:4) could reduce the proportion of amylopectin (A+B1) chains, increase the content of total starch and amylose, increase the peak viscosity and final viscosity of starch, and thus improve the cooking and eating quality of rice. Yan et al. (2018) showed that matching basal fertilizer: tillering fertilizer: panicle fertilizer=3:1:6 nitrogen management practices based on the reducing nitrogen application by 150 kg N hm^-2^ would increase the gel consistency and reduce the amylose content of rice, and ultimately improve taste quality.

Extensive research has investigated the differences in starch structure characteristics between superior and inferior grains of the same variety under different nitrogen application rates, and the changes in starch structure characteristics of whole spike grains of different varieties under various nitrogen fertilizer management practices. However, it is not clear what the effects of a high proportion of postponing N topdressing (basal fertilizer: tillering fertilizer: panicle fertilizer=3:1:6) on the starch structure characteristics of superior and inferior grains of hybrid *indica* rice with different palatability values in nitrogen fertilizer management, as well as their relationship with eating quality. Consequently, this study aims to fill this research gap by selecting two hybrid *indica* rice varieties with significant differences in taste value as the experimental materials. The objective is to investigate the effects of N fertilizer management on the starch structure and properties of superior and inferior grains within the panicles of these hybrid *indica* rice varieties. Additionally, the study aims to elucidate the relationship between the starch structure characteristics of superior and inferior grains and their respective taste qualities. We anticipate that the findings from this research endeavor will contribute to establishing a theoretical and practical basis for the cultivation of high-quality and high-yielding hybrid *indica* rice varieties.

## 2 Materials and methods

### 2.1 Test sites and experimental cultivars

Based on the experimental research conducted by our research group from 2020 to 2021 (Guo et al., 2023a), the field experiment was further improved in 2022 at the Chongzhou Experimental Base of Sichuan Agricultural University (30°70′N, 103°83′E). The preceding crop was rape, and the topsoil (0–20 cm) consisted of sandy loam soil with the following characteristics: organic matter content of 20.58 g kg^−1^, total nitrogen content of 1.53 g kg^−1^, alkaline hydrolysis nitrogen content of 102.27 mg kg^−1^, available phosphorus content of 19.42 mg kg^−1^, available potassium content of 115.45 mg kg^−1^, and a pH value of 6.61. Two medium *indica* late-maturing hybrid *indica* rice varieties (**Table 1**) with comparable growth periods and yields and significant differences in palatability were selected as the test materials (Guo et al., 2022; Guo et al., 2023b).

**Table 1:** Yield performance and taste quality of two experimental rice cultivars.

Taste value type	Cultivar	Growth period (d)	N application rate (kg N hm^–2^)	Grain yield (kg hm^–2^)	Taste value	Hardness	Stickiness
Low-taste value	F you 498	152.0	150	9397.6 a	77.69 b	3.59 a	0.20 b
High-taste value	Shuangyou 573	155.0	150	9136.1 a	85.03 a	1.91 b	0.43 a

Values within a column followed by different letters are significantly different at P < 0.05; Data are averaged in 2022 years.

### 2.2 Experimental design

The experiment employed a two-factor, completely randomized design, incorporating diverse strategies for managing N fertilizer and different varieties. Based on a stable and efficient reduction of nitrogen application rate of 150 kg N hm^-2^ (Sun et al., 2023), three N management practices were established: zero N (denoted as N_0_), local farmer practice (basal N fertilizer: tillering N fertilizer: panicle N fertilizer=7:3:0, denoted as N_1_) (Shu et al., 2021; Shu et al., 2022), postponing N topdressing (basal N fertilizer: tillering N fertilizer: panicle N fertilizer=3:1:6, denoted as N_2_). The N fertilizer (urea) was managed as follows: The base fertilizer was applied 1 d before transplanting, the tillering fertilizer was applied 7 d after transplanting, and the panicle fertilizer was applied at a 1:1 ratio during the inverse fourth-leaf and second-leaf stages. Calcium superphosphate (P_2_O_5_ 75_ kg_ hm^-2^) was applied once as basal fertilizer 1 d before transplanting, and potassium chloride (K_2_O 150 kg hm^-2^) was applied at two equal doses 1 d before transplanting and at the inverse fourth-leaf stage. on 9 April, seedlings were raised under dry conditions, and individual plants were manually transplanted on 10 May with a row spacing of 33.3 cm×16.7 cm. There were a total of six treatments, each replicated three times, with a plot area of 4 m×7 m=28.0 m^2^. A ridge (30 cm high) was constructed between adjacent plots and covered with plastic film to ensure separate irrigation and fertilization. Diseases, pests, and weeds control measures followed the management practices of high-yield fields.

### 2.3 Measurement items and methods

#### 2.3.1 Eating quality

The taste value, hardness, and stickiness of the cooked rice were measured using a Satake Rice Taste Analyzer (STA1A type, Satake, Japan) (Ma et al., 2021; Shi et al., 2022). 30 g of milled rice was weighed and placed into a matching stainless-steel tank, water was added, and the mixture was soaked for 30 min. Water was added at a rice-to-water ratio of 1:1.4, and the mouth of the tank was wrapped with filter paper, placed into a steam electric rice cooker, steamed for 30 min, and then cooled for 2 h. Rice (7 g) was weighed at room temperature and placed into a special rice press instrument to form a rice cake. It was then placed in a taste analyzer to test the taste value, hardness, and stickiness of the rice.

#### 2.3.2 Total starch content, apparent amylose content, and amylopectin content of superior and inferior grains

The grains born on the top three primary branches of rice panicles, excluding the second grain at the top, were considered superior, and the grains that were born on the three secondary branches at the base of the rice panicle, excluding the first grain at the top, were considered inferior grains (Guo et al., 2022).

The total starch content was determined using the anthrone colorimetric method (Wang et al., 2020). The apparent amylose content was determined as described by Chen et al. (2023). To 50 mg of the starch sample, 0.5 mL of anhydrous ethanol was added, followed by 4.5 mL of 1 M NaOH. The mixture was dispersed in a boiling water bath for 10 min. After cooling, the solution was adjusted to a constant volume of 50 mL. This dilution (500 μL) was placed in a centrifuge tube, and 200 μL of I_2_/KI (0.2%/2%) solution, acetic acid (1 M, 100 μL), and distilled water (9 mL) were added. Absorbance was measured at 620 nm. Amylopectin content was obtained by subtracting the apparent amylose content from the total starch content (Jiang et al., 2022a).

#### 2.3.3 Starch extraction and scanning electron microscope observation of starch granules

Starch was extracted as described by Zhang et al. (2020). Subsequently, 1 mL of anhydrous ethanol was added to the extracted 100.0 mg starch sample to prepare a starch suspension. The suspension was then dropped onto a copper conductive adhesive, evenly spread, and left at 37°C overnight. Afterward, the samples were coated with a layer of gold, and examined under a high-resolution field-emission scanning electron microscope (Zeiss Merlin Compact, Germany) to capture images of the starch granules.

#### 2.3.4 Starch granule size distribution

Referring to the method of Xiong et al. (2022a), 100 mg of starch sample was weighed and dispersed in anhydrous ethanol, and the starch granule size was determined by a Malvern laser granule size instrument (Matersizer 3000, Malvern Instruments Ltd., Worcestershire, England). And starch granules were divided into small granules (diameter < 10 μm) and large granules (diameter > 10 μm).

#### 2.3.5 Amylopectin chain length distribution

Referring to the method of Chen et al. (2023), 10 mg of starch sample was weighed, and isoamylase was used to debranch the branched starch molecules in the starch samples. The samples were analyzed by an ion chromatography system (ICS-500+; Thermo Fisher Scientific, Sunnyvale, USA). The different chain lengths of amylopectin were divided into A chain (DP 6-12), B1 chain (DP 13-24), B2 chain (DP 25-36), and B3 chain (DP>37) (Li et al., 2016).

#### 2.3.6 Starch relative crystallinity

Referring to the method of Jiang et al. (2022b), the crystallinity of starch under 40 kV and 40 mA-Cu-Kα radiation was measured by X’Pert Pro X-ray diffractometer (PANalytical, Netherlands). The scattering angle (2θ) was varied from 4° to 60° at a scanning rate of 0.02 s^-1^. And calculated starch relative crystallinity.

#### 2.3.7 Structure order of starch

Starch samples (5 mg) were thoroughly mixed with KBr at a ratio of 1:150 and pressed into slices. The samples were determined by Fourier transform infrared spectrometer NicoletiZ-10 (Thermo Fisher Scientific, Inc., Waltham, MA, USA) with 32 scans, an instrument resolution of 4 cm^-1^, and a scanning range of 400–4000 cm^-1^.

#### 2.3.8 Starch thermal properties

The thermal properties of starch were determined following the method described by Jiang et al. (2023), using a differential calorimeter (DSC 200 F3 Netzsch, Germany). A starch sample weighing 3 mg was placed in a corresponding aluminum crucible and mixed with 10 uL of deionized water. The sample was then equilibrated at 4°C for 24 h before analysis on the machine. The temperature was gradually increased at a rate of 10 °C/min, from 20°C to 100°C to assess starch gelatinization properties, and from 10°C to 90°C to evaluate starch retrogradation properties. The retrogradation percentage (%) was calculated as the retrogradation enthalpy divided by the gelatinization enthalpy, multiplied by 100 (retrogradation enthalpy/gelatinization enthalpy×100).

### 2.4 Data analysis

Analysis of variance (ANOVA) was performed using the SPSS software (version 27.0; SPSS Inc., Chicago, IL, USA). The least significant difference (LSD) was used for multiple comparisons, and Origin 2021 (OriginLab Corp., Northampton, MA) was used for plotting. Person correlation analysis was used to analyze the correlations between starch structural properties and rice eating quality at significance levels of 0.05 and 0.01.

## 3 Results

### 3.1 Eating quality

The effects of each cultivar at different grain positions and N fertilizer management on the taste value, hardness, and stickiness of superior and inferior rice grains were found to be significant level. Additionally, there was a significant interaction between these two factors in relation to the hardness of superior and inferior rice grains (**Table 2**). For each variety at different grain positions, N fertilizer management, the effect of variety differences on taste value was significantly higher than that of N fertilizer management, and the difference in inferior grains between varieties was significantly higher than that in superior grains. Compared to the low-taste variety Fyou 498, the high-taste variety Shuangyou 573 had a significant increase in the taste value of superior grains by 7.49–9.04%, and the taste value of inferior grains had a significant increase of 10.34–10.84%.

**Table 2:** Effects of postponing N topdressing on taste quality of superior and inferior grains in hybrid *indica* rice cultivars with different taste values.

Grain position	Cultivar	Treatment	Taste value	Hardness	Stickiness
Superior grain	F you 498	N_0_	85.00 ± 1.00 a	3.02 ± 0.02 c	0.25 ± 0.01 a
		N_1_	79.33 ± 1.53 c	3.36 ± 0.31 b	0.17 ± 0.03 c
		N_2_	82.80 ± 1.06 b	4.25 ± 0.21 a	0.22 ± 0.02 b
	Shuangyou 573	N_0_	92.33 ± 0.58 a	1.58 ± 0.10 b	0.49 ± 0.01 a
		N_1_	86.50 ± 0.51 c	1.91 ± 0.11 a	0.41 ± 0.01 c
		N_2_	89.00 ± 1.00 b	1.97 ± 0.11 a	0.43 ± 0.01 b
*F* value		C-SG	235.01**	629.92**	708.85**
		N	103.13**	58.78**	63.47**
		C-SG×N	1.17	20.61**	14.37**
Inferior grain	F you 498	N_0_	70.67 ± 0.58 c	3.45 ± 0.37 b	0.17 ± 0.02 b
		N_1_	72.50 ± 0.87 b	3.15 ± 0.13 c	0.20 ± 0.01 ab
		N_2_	75.83 ± 0.76 a	4.29 ± 0.17 a	0.21 ± 0.02 a
	Shuangyou 573	N_0_	78.33 ± 0.58 c	1.97 ± 0.00 b	0.42 ± 0.03 ab
		N_1_	80.00 ± 1.00 b	1.74 ± 0.04 c	0.39 ± 0.01 b
		N_2_	84.00 ± 0.50 a	2.29 ± 0.09 a	0.45 ± 0.01 a
*F* value		C-IG	1225.01**	298.15**	1134.64**
		N	79.54**	104.56**	5.96*
		C-IG×N	0.31	14.78**	3.50

N_0_, zero N; N_1_, local farmer practice; N_2_, postponing N topdressing; N, N Fertilizer treatment; C, cultivar; SG, superior grains; IG, inferior grains; C×N, cultivar and N fertilizer treatment interaction. Different lowercase letters between different N management under the same variety and grain position in the same column are significantly different at P<0.05. *, ** Significantly different at 0.05 and 0.01 probability levels.

N application significantly reduced the taste value of the superior grains of the two varieties. Compared with the N_0_ treatment, the N_1_ and N_2_ treatments significantly reduced the taste value of superior grains of the low-taste variety Fyou 498 by 6.67% and 2.59%, and the high-taste variety Shuangyou 573 by 6.31% and 3.61%, respectively. However, compared to the N_1_ treatment, the N_2_ treatment significantly increased the superior grain taste value of the low-taste variety and the high-taste variety by 4.37% and 2.89%, respectively. In contrast to the superior grains, N application treatments significantly increased the taste value of the inferior grains. Compared with N_0_, the N_1_ and N_2_ treatments significantly increased the taste value of inferior grains of the low-taste variety by 2.59% and 7.30%, and the high-taste variety by 2.13% and 7.24%, respectively, and the taste value of inferior grains significantly increased as the N topdressing ratio increased. This shows that the regulation of N fertilizer management on the taste value of inferior grains is greater than that of superior grains. At the same time, N_2_ treatment is more conducive to improving the taste value of inferior grains.

**Table 2** shows that N application treatments significantly increased the hardness of the superior grains, but significantly reduced the stickiness of superior grains in both varieties. Compared with N_0_, the N_1_ and N_2_ treatments significantly increased the hardness of superior grains of the low-taste variety by 11.26% and 40.73%, significantly decreased the stickiness by 32.00%, 12.00%; while the high-taste variety increased by 20.89%, 24.68%, and decreased by 16.33%, 12.24%. The hardness and stickiness of the inferior grains of both varieties were greatest in the N_2_ treatment. Compared with the N_2_ treatment, the N_0_ and N_1_ treatments significantly reduced the hardness of inferior grains of the low-taste variety by 19.58%, 26.57%, significantly reduced the stickiness by 19.05%, 4.76%; and the high-taste variety reduced by 13.97%, 24.02%, and 13.33%, 6.67%, respectively.

### 3.2 Rice starch and its composition

**Figure 1:**
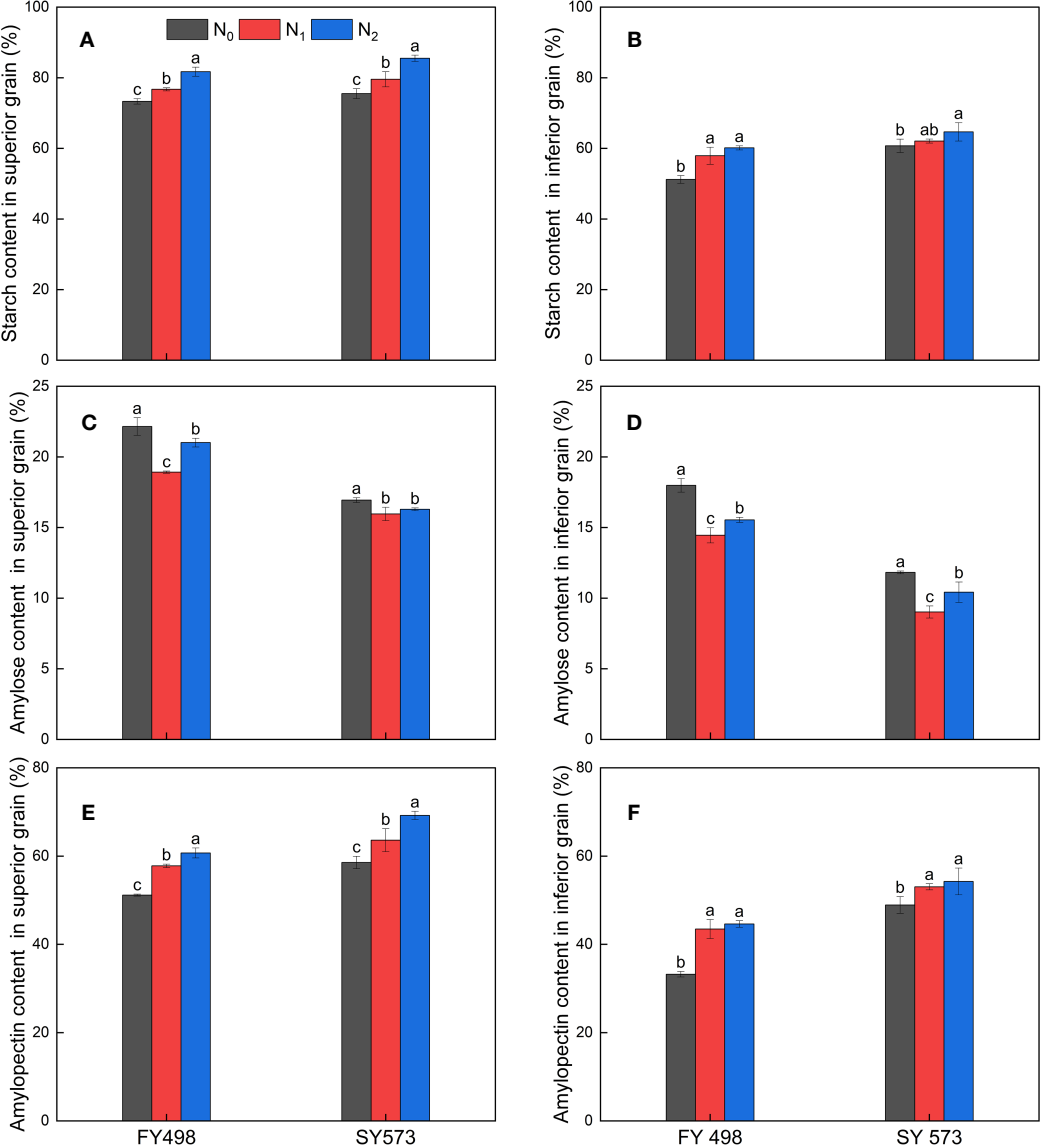
Effect of postponing N topdressing on total starch content **(A)**, amylose content **(C)**, amylopectin content **(E)** of superior grains, and total starch content **(B)**, amylose content **(D)**, and amylopectin content **(F)** of inferior grains in hybrid *indica* rice cultivars with different taste values. N_0_, Zero N; N_1_, local farmer practice; N_2_, postponing N topdressing. FY498, F you 498; SY573, Shuangyou 573. Data are presented as mean ± standard deviation. Different lowercase letters above columns indicate significant differences at *P*<0.05 among treatments at the maturity stage.

The effects of each cultivar at different grain positions on the amylose and amylopectin contents in the superior and inferior grains of rice were extremely significant (**Table 3** and **Figure 1**). The amylose content of superior and inferior grains in the high-taste variety Shuangyou 573 was significantly lower than that in the low-taste variety Fyou 498, whereas the amylopectin content was significantly higher than that in the low-taste variety Fyou 498. Compared with the low-taste variety Fyou 498, the high-taste variety Shuangyou 573 had significantly lower amylose content of 15.64–23.52% and significantly higher amylopectin content of 10.07–14.49% in the superior grains; while the inferior grains had significantly lower amylose content of 32.95–37.58% and significantly higher amylopectin content of 21.64–47.24%.

**Table 3:** Analysis of variance for starch content characteristics of superior and inferior grains in hybrid *indica* rice cultivars with different taste values (*F* values).

Grain position	Treatment	Starch content	Amylose content	Amylopectin content
Superior grain	C-SG	15.97	1423.16**	134.13**
	N	83.49**	71.48**	82.44**
	C-SG×N	0.62	22.19**	1.47
Inferior grain	C-IG	370.63**	510.04**	501.81**
	N	24.03**	76.50**	100.58**
	C-IG×N	5.44*	2.18	15.07**

C, cultivar; SG, superior grains; IG, Inferior grains; N, N Fertilizer treatment; C×N, cultivar and N fertilizer treatment interaction.

*, ** Significantly different at 0.05 and 0.01 probability levels.

N fertilizer management had significant effects on the total starch, amylose, and amylopectin contents of superior and inferior grains. N application treatments significantly increased the total starch and amylopectin contents of the superior grains in both varieties but significantly decreased the amylose content. Compared with N_0_, the N_1_ and N_2_ treatments significantly increased the total starch content of superior grains of the low-taste variety by 4.68%, and 11.49%, and significantly increased amylopectin content by 13.00%, and 18.69%, but significantly decreased amylose by 14.58%, 5.15%; while the high-taste variety increased by 5.38%, 13.25% and 8.64%, 18.21%, and decreased by 5.79%,3.84%. In line with the trend of superior grains, N application treatments significantly increased the total starch and amylopectin content and significantly decreased the amylose content of inferior grains in both varieties. Compared with N_0_, N_1_ and N_2_ significantly increased the total starch content of inferior grains of low-taste variety by 13.13%, 17.48%, significantly increased amylopectin content by 30.86%, 34.30%, significantly decreased amylose content by 19.63%, 13.57%; while the high-taste variety increased by 2.21%, 6.51%, and 8.47%,10.94%, and decreased by 23.69%, 11.84%, respectively.

### 3.3 Scanning electron microscope observation of starch granules

**Figure 2:**
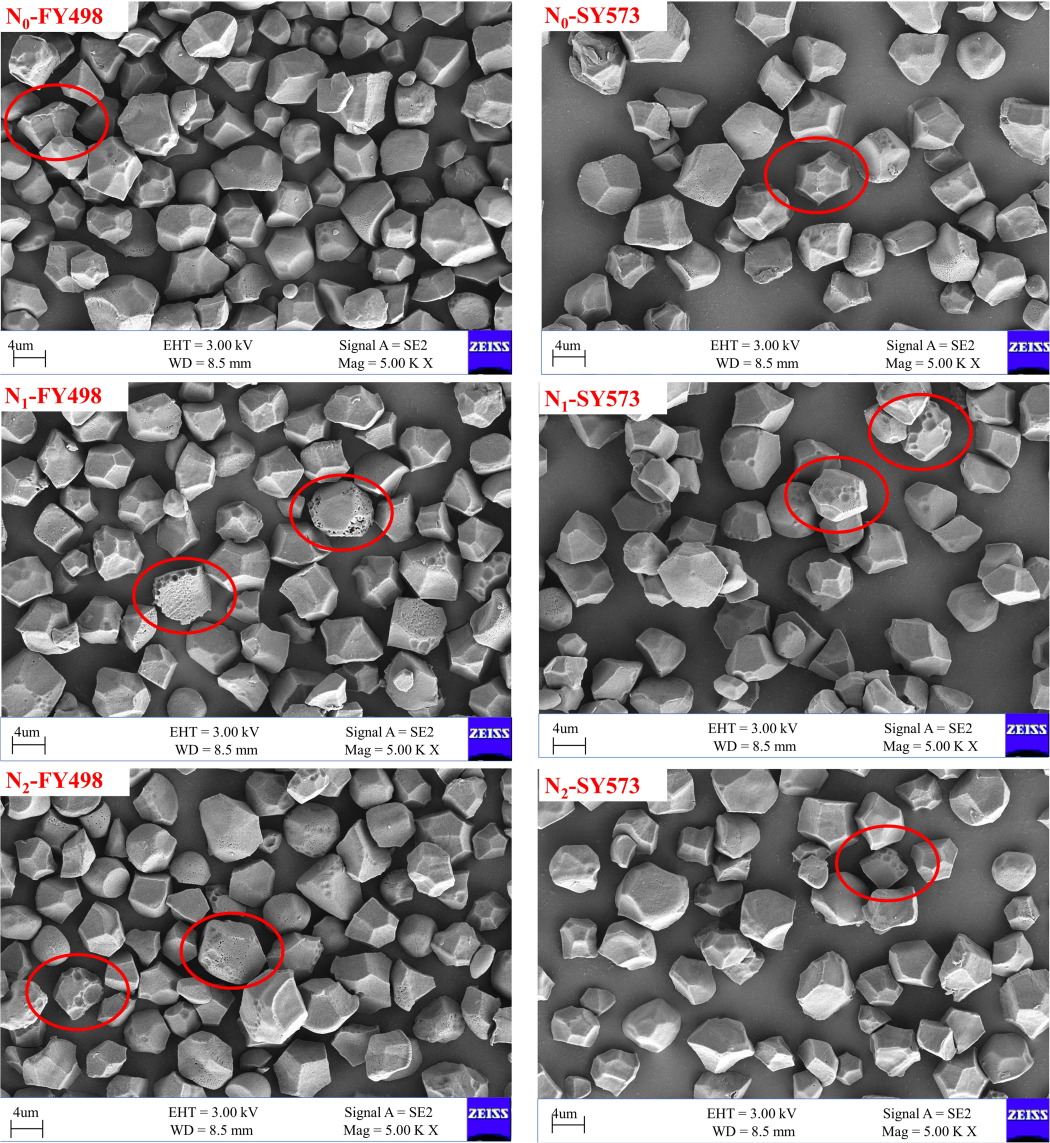
Scanning electron microscopic (SEM) of superior starch in hybrid *indica* rice cultivars with different taste values under postponing N topdressing. N_0_, Zero N; N_1_, local farmer practice; N_2_, postponing nitrogen topdressing; FY498, F you 498; SY573, Shuangyou 573.

**Figure 3:**
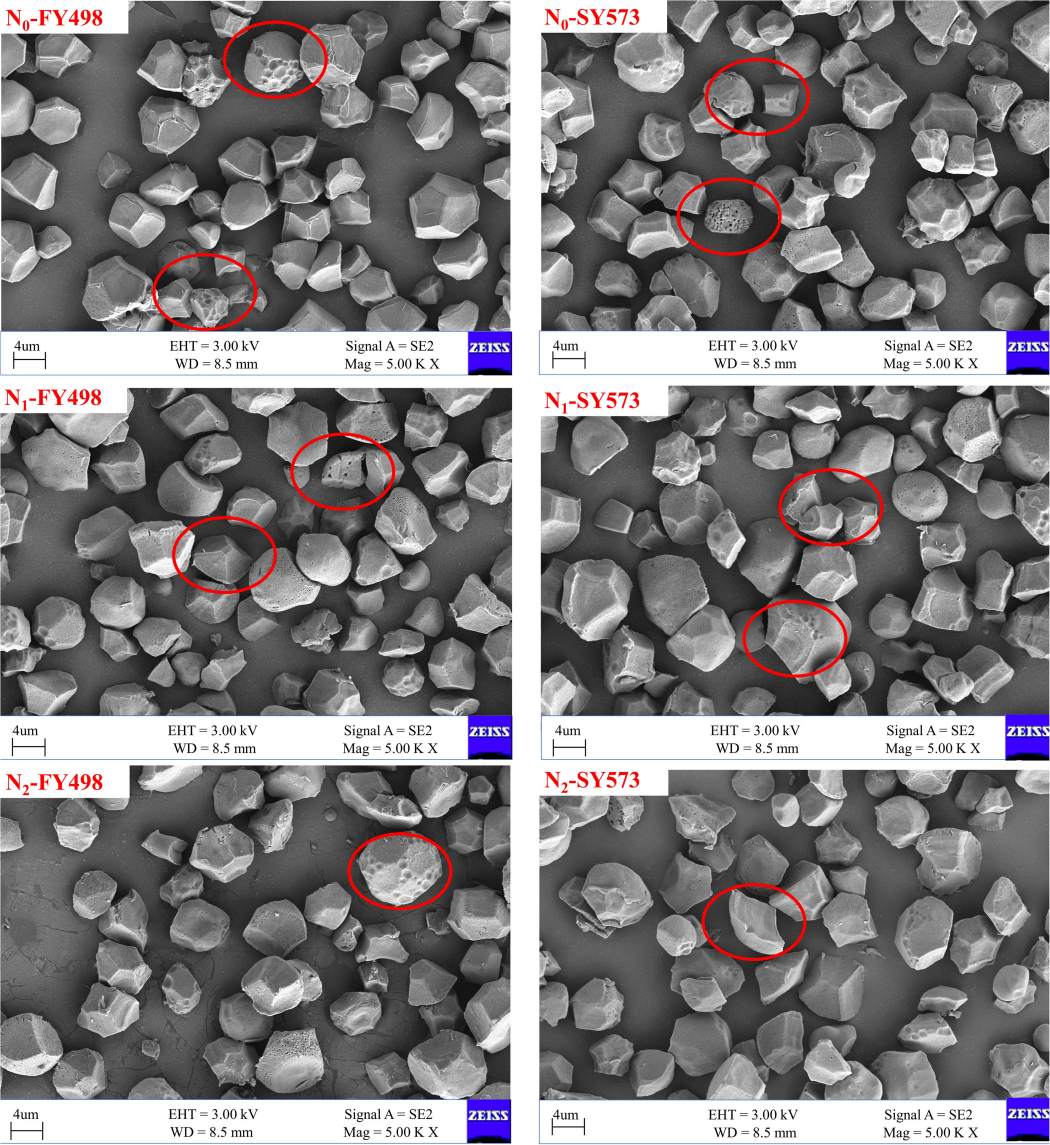
Scanning electron microscopic (SEM) of inferior starch in hybrid *indica* rice cultivars with different taste values under postponing N topdressing. N_0_, Zero N; N_1_, local farmer practice; N_2_, postponing N topdressing; FY498, F you 498; SY573, Shuangyou 573.

As shown in **Figures 2**, **3**, compared with the low-flavored variety Fyou 498, the starch granule surfaces of the superior and inferior grains of the high-flavored variety Shuangyou 573 were smoother, with fewer holes and pits and more complete and uniform starch granules. In addition, the difference between the inferior grains was more obvious. Under each N treatment, the starch granules of the superior and inferior grains of both varieties exhibited irregular polygons.

Compared with the N_0_ treatment, there were more holes and pits on the surface of superior grain starch granules and more small granule starch content in the two varieties under N application treatments. In particular, the starch content of small granules was obviously increased under the N_2_ treatment. The starch granules of the inferior grains under each N fertilizer treatment were more uneven than those of the superior grains, and the surfaces of the starch granules were more uneven and cracked. Compared to the N_0_ treatment, the N_1_ and N_2_ treatments had more complete starch granules and fewer broken starch granules in the inferior grains. The application of nitrogen fertilizer deteriorated the starch granule traits of superior grains but optimized the starch granule traits of inferior grains.

### 3.4 Starch granule size distribution

As shown in **Table 4**, the effects of each variety at different grain positions and N fertilizer management on the percentage of superior and inferior grains, small and large starch granules, and starch volume mean diameter (D[4,3]) reached significant or highly significant levels, and the interaction effect of the two factors was highly significant. The N application treatments significantly increased the proportion of small starch granules and reduced the proportion of large starch granules and the D[4,3] of superior grains in both varieties. Compared with N_0_, the N_1_ and N_2_ treatments significantly increased the proportion of small starch granules in superior grains of the low-taste variety Fyou 498 by 9.40%, 10.65%, and significantly decreased the proportion of larger starch granules and D[4,3] by 34.14%, 38.68% and 2.40%, 4.40%, and the high-taste variety Shuangyou 573 increased by 5.42%, 5.69%, and decreased by 12.94%, 13.58%, and 3.03%, 4.92%, respectively. Compared with N_1_, N_2_ treatment further increased the proportion of small starch granules in the superior grains and reduced the proportion of large starch granules and D[4,3] of the two varieties. Among them, the increase of superior grains in small starch granules and the decrease in large starch granules in high-taste varieties were lower than those in low-taste varieties. Consistent with the trend in superior grains, both the N_1_ and N_2_ treatments significantly increased the proportion of small starch granules and significantly reduced the proportion of large starch granules in inferior grains of both varieties. Compared with the N_0_ treatment, N_1_ and N_2_ treatments significantly increased the proportion of small starch granules in inferior grains of low-taste variety by 1.18% and 2.02%, decreased the proportion of large starch granules by 5.70% and 9.71%; while the high-taste variety increased by 15.95% and 16.75%, and decreased by 35.12% and 36.88%. Meanwhile, the proportion of small starch granules in the inferior grains increased, and the proportion of large starch granules decreased as the proportion of postponed N topdressing. The effects of N management on inferior grain D[4,3] differed between the two varieties. The low-taste variety Fyou 498 inferior grains D[4,3] was the lowest under the N_1_ treatment, significantly lower by 13.69% compared to the N_0_ treatment and 11.33% compared to the N_2_ treatment; while the high-taste variety Shuangyou 573 inferior grains D[4,3] was the highest in the N_1_ treatment, higher by 2.96% compared to the N_0_ treatment and 5.70% compared to the N_2_ treatment. The results showed that the N application treatments increased the percentage of small starch granules and decreased the percentage of large starch granules and D[4,3]. And the percentage of small starch granules increased significantly, and the percentage of large starch granules and D[4,3] decreased significantly with the proportion of N postponed topdressing. Besides, the increase of small starch granules and the decrease of large starch granules in superior grains of high-taste variety Shuangyou 573 under N_2_ treatment were lower than those of low-taste variety Fyou 498. However, the increase of small starch granules and the decrease of large starch granules in inferior grains were higher than those of low-taste variety, which may be an important reason for Shuangyou 573 under N_2_ treatment to maintain the high taste value of inferior grains.

**Table 4:** Effects of postponing N topdressing on starch granule size distribution of superior and inferior grains in hybrid *indica* rice cultivars with different taste values.

Grain position	Cultivar	Treatment	SSG < 10 μm (%)	LSG > 10 μm (%)	D [4,3] (μm)
Superior grain	F you 498	N_0_	78.41 ± 0.91 c	21.59 ± 1.77 a	2.50 ± 0.11 a
		N_1_	85.78 ± 0.78 b	14.22 ± 0.69 b	2.44 ± 0.10 b
		N_2_	86.76 ± 1.18 a	13.24 ± 1.09 b	2.39 ± 0.10 c
	Shuangyou 573	N_0_	70.47 ± 1.03 b	29.53 ± 2.31 a	2.64 ± 0.08 a
		N_1_	74.29 ± 1.84 a	25.71 ± 0.85 b	2.56 ± 0.07 b
		N_2_	74.48 ± 1.44 a	25.52 ± 1.45 b	2.51 ± 0.09 c
F value		C-SG	1454.71**	2684.73**	88.40*
		N	676.48**	166.28**	270.92**
		C-SG×N	80.67**	19.83**	59.29**
Inferior grain	F you 498	N_0_	82.81 ± 1.06 c	17.19 ± 1.15 a	2.63 ± 0.05 a
		N_1_	83.79 ± 1.27 b	16.21 ± 1.17 b	2.27 ± 0.04 b
		N_2_	84.48 ± 1.22 a	15.52 ± 1.42 c	2.56 ± 0.19 a
	Shuangyou 573	N_0_	68.76 ± 1.47 c	31.24 ± 1.98 a	2.70 ± 0.21 ab
		N_1_	79.73 ± 1.20 b	20.27 ± 1.10 b	2.78 ± 0.17 a
		N_2_	80.28 ± 1.38 a	19.72 ± 1.37 b	2.63 ± 0.12 b
F value		C-IG	5972.38**	2961.85**	26.19*
		N	4012.50**	549.71**	12.54**
		C-IG×N	4312.35**	590.79**	15.30**

N_0_, Zero N; N_1_, local farmer practice; N_2_, postponing N topdressing; SSG, small starch granules; LSG, large starch granules; D[4,3], starch volume mean diameter; N, N Fertilizer treatment; C, cultivar; SG, superior grains; IG, inferior grains; C×N, cultivar and N fertilizer treatment interaction. Different lowercase letters between different N management under the same variety and the same grain position in the same column are significantly different at P<0.05. *, ** Significantly different at 0.05 and 0.01 probability levels.

### 3.5 Amylopectin chain length distribution

**Figure 4:**
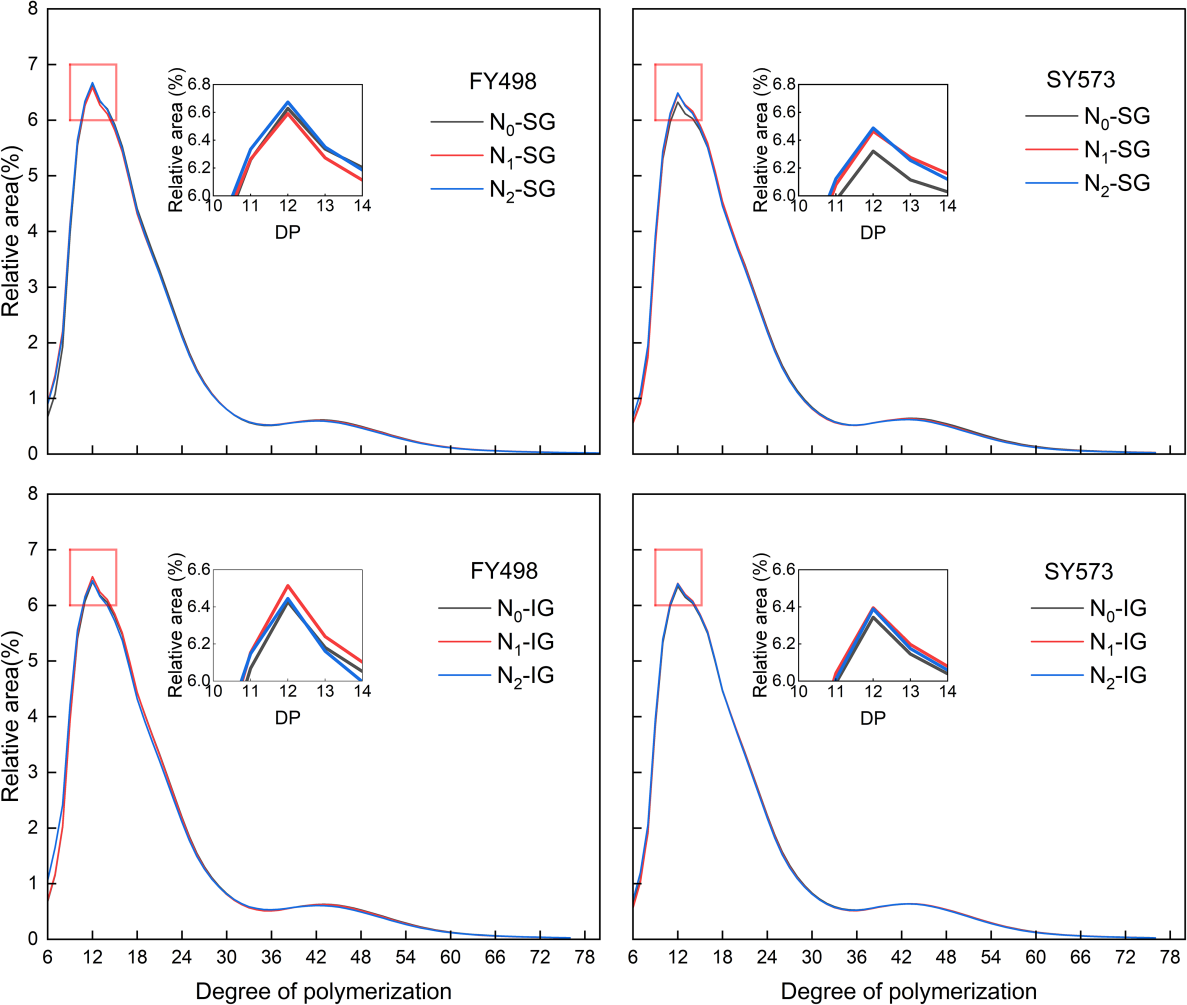
Effect of postponing N topdressing on amylopectin chain length distribution of superior grains and inferior grains in hybrid *indica* rice cultivars with different taste values. N_0_, Zero N; N_1_, local farmer practice; N_2_, postponing N topdressing. FY498, F you 498; SY573, Shuangyou 573.

As shown in **Table 5**, the varieties of different grain positions and N fertilizer management had significant effects on the amylopectin chain length distribution index in the superior and inferior grains, and the interaction effect of the two factors had significant effects on the proportions of the A, B1, B3 chains, and (B2+B3) in the superior and inferior grains. The distribution trend of the amylopectin chain length in the superior and inferior grains of low-taste and high-taste variety was consistent and bimodal under each N fertilizer treatment, reaching a peak at DP=12 (**Figure 4**). This may be related to the fact that the distribution of chain length between superior and inferior grains is mainly determined by genetic background (Jiang et al., 2022a). The N application treatments increased the proportion of A chain and (A+B1) chain and decreased the proportion of B2 chain, B3 chain, and (B2+B3) chain in superior and inferior grains of the low-taste and high-taste variety. Among them, compared with N_0_, under N_1_ and N_2_ treatments, the proportion of A chain in superior grains of the low-taste variety increased by 4.06% and 4.13%, and the B3 chain decreased by 1.10% and 3.67%, while the proportion of A chain of the high-taste variety increased by 0.81% and 4.13%, and the B3 chain decreased by 3.75% and 5.88%, respectively. Meanwhile, the proportion of (B2+B3) in inferior grains of two varieties decreased by 1.69%, 2.84%, and 1.22%, 2.19%. Similarly, compared with N_1_, the N_2_ treatment further increased the proportion of A chain and (A+B1) chain of superior grains in two varieties and further significantly reduced the proportion of B3 chain and (B2+B3) chain. Among them, the proportion of the B3 chain was significantly reduced by 2.59% and 2.21%. Significantly, inferior grains trend in line with superior grains. Among them, the proportion of A chain in inferior grains of low-taste and high-taste variety was significantly reduced by 5.84% and 1.86%, and the proportion of B3 chain was significantly reduced by 2.41% and 1.30%.

**Table 5:** Effects of postponing N topdressing on amylopectin chain length distribution of superior and inferior grains in hybrid *indica* rice cultivars with different taste values.

Grain position	Cultivar	Treatment	A (DP 6-12) (%)	B1(DP 13-24) (%)	B2(DP 25-36) (%)	B3 (DP >37) (%)	A+B1 (%)	B2+B3 (%)
Superior grain	F you 498	N_0_	26.13 ± 0.35 b	52.05 ± 0.10 a	10.91 ± 0.07 a	10.91 ± 0.13 a	78.18 ± 0.45 c	21.82 ± 0.17 a
		N_1_	27.19 ± 0.15 a	51.13 ± 0.32 c	10.89 ± 0.10 a	10.79 ± 0.20 b	78.32 ± 0.48 b	21.68 ± 0.11 b
		N_2_	27.21 ± 0.23 a	51.46 ± 0.21 b	10.82 ± 0.12 b	10.51 ± 0.16 c	78.67 ± 0.43 a	21.33 ± 0.26 c
	Shuangyou 573	N_0_	24.71 ± 0.19 c	52.19 ± 0.10 b	11.37 ± 0.08 a	11.73 ± 0.15 a	76.90 ± 0.29 c	23.10 ± 0.13 a
		N_1_	24.91 ± 0.09 b	52.70 ± 0.20 a	11.10 ± 0.13 b	11.29 ± 0.12 b	77.61 ± 0.29 b	22.39 ± 0.15 b
		N_2_	25.73 ± 0.14 a	52.10 ± 0.17 b	11.13 ± 0.15 b	11.04 ± 0.07 c	77.83 ± 0.31 a	22.17 ± 0.12 c
F value		C-SG	837.67**	647.21**	588.01**	456.34**	108.77**	3754.63**
		N	265.29**	23.87**	50.33**	310.91**	1997.58**	504.97**
		C-SG×N	54.74**	107.23**	24.57**	32.68**	349.08**	88.24**
Inferior grain	F you 498	N_0_	25.74 ± 0.10 b	51.76 ± 0.15 a	11.09 ± 0.15 a	11.41 ± 0.03 a	77.50 ± 0.25 b	22.50 ± 0.18 a
		N_1_	26.01 ± 0.07 b	51.87 ± 0.14 a	10.93 ± 0.06 b	11.19 ± 0.10 b	77.88 ± 0.20 ab	22.12 ± 0.16 b
		N_2_	27.53 ± 0.10 a	50.61 ± 0.51 b	10.94 ± 0.06 b	10.92 ± 0.04 c	78.14 ± 0.61 a	21.86 ± 0.10 c
	Shuangyou 573	N_0_	25.16 ± 0.60 b	51.98 ± 0.12 a	11.28 ± 0.11 a	11.58 ± 0.07 a	77.14 ± 0.72 b	22.86 ± 0.19 a
		N_1_	25.29 ± 0.07 b	52.13 ± 0.24 a	11.06 ± 0.16 b	11.52 ± 0.10 b	77.42 ± 0.30 ab	22.58 ± 0.26 b
		N_2_	25.76 ± 0.09 a	51.88 ± 0.32 a	10.99 ± 0.10 b	11.37 ± 0.10 c	77.64 ± 0.41 a	22.36 ± 0.20 c
F value		C-IG	117.76**	637.98**	41.07*	270.77**	38.18*	118.53**
		N	54.30**	35.83**	45.75**	214.84**	10.08**	310.36**
		C-IG×N	14.02**	19.46**	3.96	34.15**	0.16	4.93*

N_0_, Zero N; N_1_, local farmer practice; N_2_, postponing N topdressing; DP, degree of polymerization; N, N Fertilizer treatment; C, cultivar; SG, superior grains; IG, inferior grains; C×N, cultivar and N fertilizer treatment interaction. Different lowercase letters between different N management under the same variety and the same grain position in the same column are significantly different at P<0.05. *, ** Significantly different at 0.05 and 0.01 probability levels.

### 3.6 Relative crystallinity and ATR-FTIR spectrum of starch

As shown in **Table 6**, each variety at different grain positions had a significant effect on the relative crystallinity and the 1045/1022 cm^-1^ peak intensity ratio of starch. Comparing the high-taste variety Shuangyou 573 with the low-taste variety Fyou 498, there was a significant increase of 1.73–7.67% and 3.04–3.62% in the relative crystallinity and 1045/1022 cm^-1^ value of superior grains.

**Table 6:** Effects of postponing N topdressing on relative crystallinity and the infrared ratio of superior and inferior grains in hybrid *indica* rice cultivars with different taste values.

Grain position	Cultivar	Treatment	Relative crystallinity (%)	Infrared ratio	
				1045/1022 (cm^-1^)	1022/995 (cm^-1^)
Superior grain	F you 498	N_0_	22.95 ± 0.12 a	0.729 ± 0.001 a	1.292 ± 0.013 c
		N_1_	22.68 ± 0.10 b	0.718 ± 0.008 b	1.301 ± 0.004 b
		N_2_	22.49 ± 0.20 c	0.691 ± 0.005 c	1.311 ± 0.010 a
	Shuangyou 573	N_0_	24.55 ± 0.26 a	0.753 ± 0.004 a	1.274 ± 0.009 c
		N_1_	24.42 ± 0.30 a	0.744 ± 0.001 b	1.280 ± 0.012 b
		N_2_	22.88 ± 0.13 b	0.712 ± 0.002 c	1.353 ± 0.009 a
*F* value		C-SG	506.74**	412.93**	6.39
		N	354.39**	363.02**	400.31**
		C-SG×N	152.37**	1.57	182.44**
Inferior grain	F you 498	N_0_	22.71 ± 0.05 c	0.712 ± 0.004 c	1.353 ± 0.008 a
		N_1_	22.91 ± 0.02 a	0.744 ± 0.001 a	1.280 ± 0.003 c
		N_2_	22.83 ± 0.02 b	0.734 ± 0.003 b	1.295 ± 0.007 b
	Shuangyou 573	N_0_	22.86 ± 0.10 c	0.732 ± 0.002 b	1.277 ± 0.006 b
		N_1_	25.26 ± 0.11 a	0.748 ± 0.003 a	1.274 ± 0.011 b
		N_2_	23.70 ± 0.18 b	0.746 ± 0.001 a	1.287 ± 0.010 a
*F* value		C-IG	392.26**	1487.49**	381.73**
		N	2190.62**	525.60**	307.25**
		C-IG×N	1594.00**	76.69**	319.95**

N_0_, Zero N; N_1_, local farmer practice; N_2_, postponing N topdressing; N, N Fertilizer treatment; C, cultivar; SG, superior grains; IG, inferior grains; C×N, cultivar and N fertilizer treatment interaction. Different lowercase letters between different N management under the same variety and the same grain position in the same column are significantly different at P<0.05. *, **Significantly different at 0.01 probability levels.

**Figure 5:**
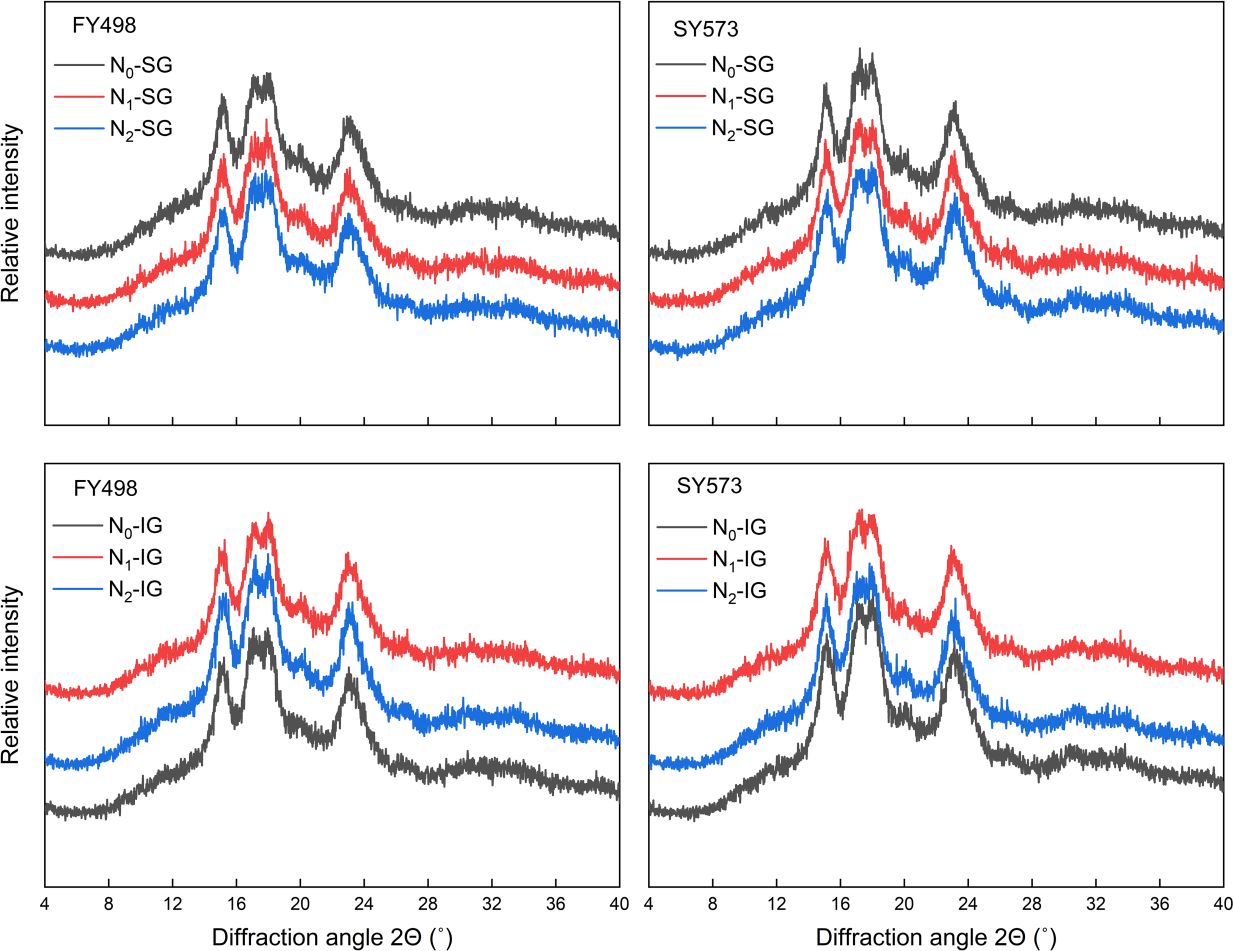
Effects of postponing N topdressing on X-ray diffraction patterns of superior and inferior grains in hybrid *indica* rice cultivars with different taste values. N_0_, Zero N; N_1_, local farmer practice; N_2_, postponing N topdressing. FY498, F you 498; SY573, Shuangyou 573. SG, superior grains; IG, inferior grains.

Under each treatment, the X-ray diffraction pattern of the superior and inferior starch grains of the two varieties showed two obvious strong peaks at 15° and 23° and two continuous double peaks near 17° and 18°, which are typical A-type crystallization peaks (**Figure 5**). This indicates that N fertilizer management does not change the crystal structure of starch. Further analysis revealed that N application treatments had a significant impact on the relative crystallinity and infrared ratio (IR) value of superior and inferior grains. Moreover, N application treatments reduced the relative crystallinity and 1045/1022 cm^-1^ value of the low-taste variety and high-taste variety of superior grains but increased the 1022/995 cm^-1^ value. Among them, compared with N_0_, under N_2_ treatment, the relative crystallinity and the 1045/1022 cm^-1^ value of the low-taste variety decreased by 2.00% and 5.44%, and the high-taste variety decreased by 6.80% and 5.44%, respectively. In contrast to the superior grains, N application treatments significantly increased the relative crystallinity and 1045/1022 cm^-1^ peak intensity ratio of the inferior grains. Compared to N_0_, the N_1_ and N_2_ treatments increased the relative crystallinity of inferior grains of the two varieties, with a significant increase of 10.50% and 3.67% of the high-taste variety. Besides, in the N_1_ and N_2_ treatments, the 1045/1022 cm^-1^ value of the inferior grains in the low-taste variety significantly increased by 4.49% and 3.09%, and the high-taste variety significantly increased by 2.19% and 1.91%, respectively. The effects of N fertilizer treatment on the 1022/995 cm^-1^ peak intensity ratio of inferior grains differed between the two varieties. The low-taste variety exhibited the highest 1022/995 cm^-1^ peak intensity ratio under the N_0_ treatment, significantly increasing by 5.40% and 4.29% compared to the N_1_ and N_2_ treatments, respectively. In contrast, the high-taste variety was the highest under the N_2_ treatment, increasing by 1.01%, compared to the N_1_ treatments. Compared with N_1_, the N_2_ treatment significantly reduced the relative crystallinity and the 1045/1022 cm^-1^ value and significantly increased the 1022/995 cm^-1^ value of superior grains and inferior grains in both varieties. In particular, the high-taste variety of superior grains and inferior grains showed the greatest reduction in relative crystallinity, 6.31%, and 6.18%, respectively. This indicates that application of N fertilizer reduced the relative crystallinity and order degree of starch granules in superior grains, whereas the opposite trend was observed in inferior grains. Increasing the proportion of N topdressing ratio moderately reduced the relative crystallinity and ordered structure of starch granules, thereby changing their stability of starch granules.

### 3.7 Starch thermal properties

The cultivars of different grain positions had significant or extremely significant effects on the onset temperature, peak temperature, termination temperature, retrogradation enthalpy, and retrogradation percentage of starch in the superior and inferior grains. N application management had extremely significant effects on the indicators of starch thermal properties in superior and inferior grains, and the interaction effect of the two factors on each index was extremely significant (**Table 7**). Under each variety of different grain positions, and N fertilizer treatments, the effects of the varieties on the starch onset, peak, and termination temperatures were significantly higher than those of nitrogen fertilizer. Compared with the low-taste variety Fyou 498, the onset, peak, and termination temperatures of superior grains starch of the high-taste variety Shuangyou 573 were significantly increased by 3.47–7.25%, 4.13–5.54%, and 2.69–3.93%, respectively; while the onset, and termination temperatures of inferior grains starch were significantly increased by 3.88–7.25%, and 1.37–3.79%, respectively.

**Table 7:** Effects of postponing N topdressing on starch thermal properties of superior and inferior grains in hybrid *indica* rice cultivars with different taste values.

Grain position	Cultivar	Treatment	ΔHgel (J g^–1^)	To (°C)	Tp (°C)	Tc (°C)	ΔHret (J g^–1^)	R(%)
Superior grain	F you 498	N_0_	12.28 ± 0.21 a	69.10 ± 0.16 b	76.70 ± 0.60 a	83.90 ± 0.02 a	7.36 ± 0.38 b	59.93 ± 0.81 b
		N_1_	12.33 ± 0.17 a	68.60 ± 0.42 c	76.00 ± 0.57 b	83.30 ± 0.06 b	9.19 ± 0.13 a	74.53 ± 1.10 a
		N_2_	12.12 ± 0.20 b	69.50 ± 0.50 a	76.60 ± 0.60 a	83.20 ± 0.13 b	5.82 ± 0.35 c	48.02 ± 0.51 c
	Shuangyou 573	N_0_	11.80 ± 0.20 c	74.50 ± 0.25 a	81.20 ± 0.46 a	87.10 ± 0.24 a	1.63 ± 0.50 b	13.81 ± 0.43 b
		N_1_	12.05 ± 0.10 a	73.80 ± 0.21 b	79.70 ± 0.32 c	85.60 ± 0.38 c	2.92 ± 0.28 a	24.23 ± 0.32 a
		N_2_	11.89 ± 0.13 b	72.00 ± 0.18 c	79.90 ± 0.20 b	86.60 ± 0.36 b	1.44 ± 0.60 b	12.11 ± 0.17 c
*F* value		C-SG	130.68**	2659.30**	635.71**	400.79**	3033.80**	23349.01**
		N	37.79**	101.41**	215.84**	377.08**	391.66**	11151.95**
		C-SG×N	17.12**	239.67**	64.98**	116.63**	61.17**	1591.87**
Inferior grain	F you 498	N_0_	13.04 ± 0.20 a	69.30 ± 0.69 a	79.50 ± 0.17 a	86.40 ± 0.20 a	6.87 ± 0.17 a	52.68 ± 0.37 b
		N_1_	11.66 ± 0.13 b	68.70 ± 0.56 b	79.30 ± 0.07 a	86.20 ± 0.06 b	5.24 ± 0.27 b	44.94 ± 0.20 c
		N_2_	11.53 ± 0.31 b	66.50 ± 0.59 c	78.80 ± 0.15 b	86.20 ± 0.12 b	6.37 ± 0.30 a	55.25 ± 1.01 a
	Shuangyou 573	N_0_	13.58 ± 0.36 a	72.10 ± 0.21 a	80.20 ± 0.36 b	87.60 ± 0.22 c	10.29 ± 0.10 a	75.77 ± 1.30 a
		N_1_	12.19 ± 0.10 b	72.40 ± 0.23 a	81.40 ± 0.57 a	88.70 ± 0.30 b	4.82 ± 0.90 c	39.54 ± 0.71 c
		N_2_	10.47 ± 0.40 c	71.70 ± 0.64 b	81.60 ± 0.64 a	89.60 ± 0.14 a	6.01 ± 0.51 b	57.40 ± 0.63 b
*F* value		C-IG	0.01	710.98**	67.57*	1929.10**	35.27*	1051.70**
		N	466.54**	147.48**	32.87**	214.41**	238.79**	4721.27**
		C-IG×N	73.18**	69.41**	142.72**	323.82**	88.17**	2073.09**

N_0_, Zero N; N_1_, local farmer practice; N2, postponing N topdressing; ΔHgel, gelatinization enthalpy; To, onset temperature; T_P_, peak of gelatinization temperature; T_C_, conclusion temperature; ΔHret, retrogradation enthalpy; R, Retrogradation percentage. R (%) = ΔHret/ΔHgel × 100%. N, N Fertilizer treatment; C, cultivar; SG, superior grains; IG, inferior grains; C×N, cultivar and N fertilizer treatment interaction. Different lowercase letters between different N management under the same variety and the same grain position in the same column are significantly different at P<0.05. *, ** Significantly different at 0.05 and 0.01 probability levels.

Under different N application treatments, the N_1_ treatment increased the gelatinization enthalpy of the superior grains, while the N_2_ treatment decreased the gelatinization enthalpy of both varieties. Moreover, compared with N_0_, N application reduced the peak and termination temperatures of the superior grain of the two varieties. Inconsistent with the trend of superior grains, N application treatments reduced the gelatinization enthalpy, and retrogradation enthalpy of inferior grains of the two varieties; reduced the starch peak and termination temperature of inferior grains of the low-taste variety Fyou 498; and increased the starch peak and termination temperature of inferior grains of the high-taste variety Shuangyou 573. Among them, compared with N_0_, in the N_1_ and N_2_ treatments, the gelatinization enthalpy in inferior grains of low-taste variety decreased by 10.58% and 11.58%, and retrogradation enthalpy decreased by 23.73% and 7.28%, while the high-taste variety decreased by 10.24% and 22.90%, 41.59%, and 53.16%, respectively. The results showed that the N application improved the gelatinization and retrogradation properties of inferior grains by reducing the gelatinization enthalpy and retrogradation enthalpy of the inferior grains of the two varieties. Increasing the proportion of postponed N topdressing reduced the gelatinization enthalpy of rice, but increased its retrogradation enthalpy.

### 3.8 Relationship between starch structure properties and eating quality of superior and inferior grains

**Figure 6:**
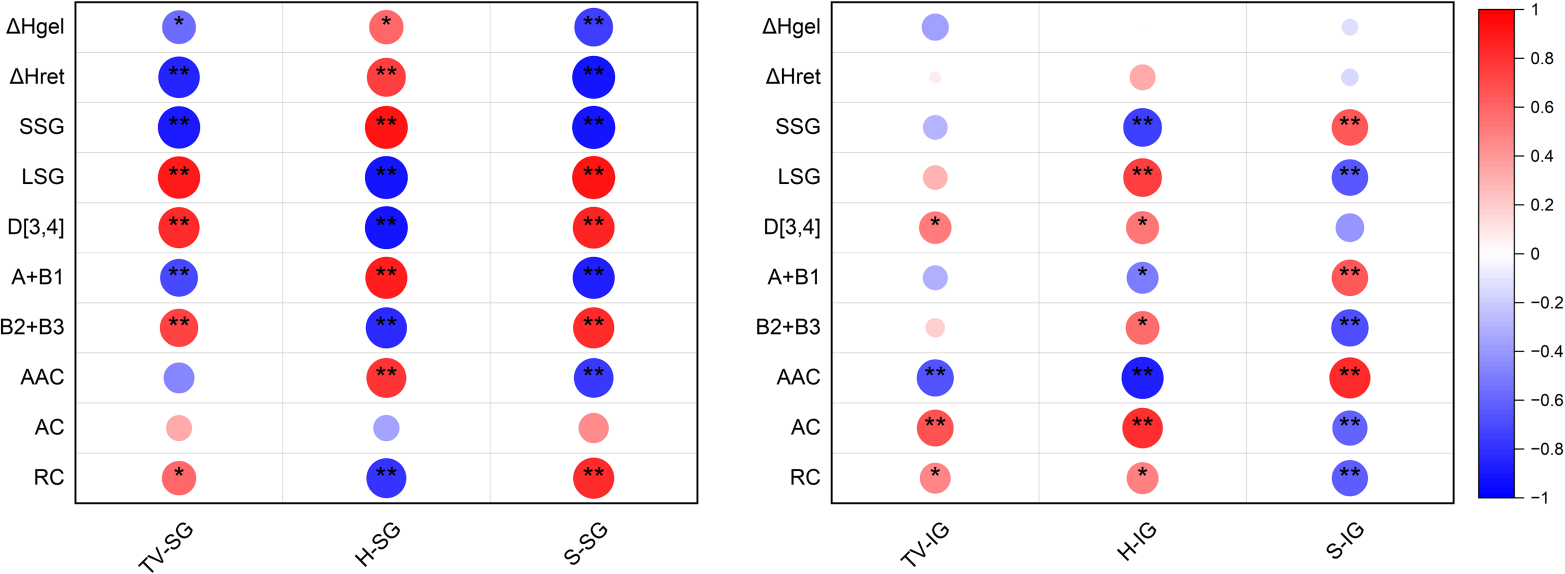
Heat map of person correlation in eating quality indicators, starch structure and properties of superior and inferior grains in hybrid *indica* rice cultivars. TV, taste value; H, Hardness; S, stickiness; SG, superior grains; IG, inferior grains; ΔHgel, gelatinization enthalpy; ΔHret, retrogradation enthalpies; SSG, small starch granules; LSG, large starch granules; D[4,3], starch volume mean diameter; (A+B1), amylopectin chains with DP 6-12 + DP 13-24; (B2+B3), amylopectin chains with DP 25-36 + DP>36. AAC, apparent amylose content; AC, amylopectin content; RC, relative crystallinity. *, ** Significantly different at 0.05 and 0.01 probability levels.

As shown in **Figure 6**, under different varieties and N fertilizer management, the taste value and stickiness of superior grains were significantly or extremely significantly positively correlated with the proportion of large starch granules, D[4,3], the proportion of (B2+B3) chains, and relative crystallinity, and significantly or extremely significantly negatively correlated with the gelatinization enthalpy, retrogradation enthalpy, proportion of small starch granules, the proportion of (A+B1) chain, and amylose content. The superior grain hardness exhibited an opposite trend to that of stickiness.

The taste value of inferior grains was significantly or extremely significantly positively correlated with D[4,3], amylopectin content, and relative crystallinity, and significantly negatively correlated with amylose content. Hardness was significantly or extremely significantly positively correlated with the proportion of large-granule starch, D[4,3], (B2+B3) chain, amylopectin content, and relative crystallinity, and significantly negatively correlated with the proportion of small-granule starch, (A+B1) chain, and amylose content. Stickiness was significantly positively correlated with the proportion of small granule starch, the proportion of (A+B1) chains, and relative crystallinity and was significantly negatively correlated with the proportion of large granule starch, the proportion of (B2+B3) chains, amylopectin content, and relative crystallinity.

## 4 Discussion

### 4.1 Effects of N fertilizer management on starch structure and thermal properties of superior and inferior grains

The quality of rice starch is regulated by genetic factors and agronomic cultivation practices. Rice varieties with different genetic backgrounds exhibit different starch structures and properties. In this study, the varieties had significant effects on the fine structure and thermal properties of starch in superior and inferior grains. Compared with the low-taste variety Fyou 498, the large proportion of large starch granules and (B2+B3) chains in the superior and inferior grains of the high-taste variety Shuangyou 573 is an important physiological basis for the high relative crystallinity of starch, order degree of starch granules, and range of starch gelatinization temperatures (onset, peak, and termination temperature) of the high-taste variety Shuangyou 573, which further complements and improves the results of previous studies (Peng et al., 2021; Gao et al., 2022; Xiao et al., 2022). This study also showed that the high-taste variety Shuangyou 573 significantly reduced the starch retrogradation enthalpy of the superior and inferior grains, which may have been caused by the low amylose content of Shuangyou 573.

N fertilizer in agronomic cultivation measures has a significant effect on the regulation of starch structure and physicochemical properties of superior and inferior grains (Jiang et al., 2022b). Previous studies have shown that N fertilization affects the morphology and size of starch granules. Optimized N application improved starch granule traits, while excessive postponement of N topdressing resulted in more small starch granules, which may be related to the formation of starch granules at different stages of grain filling (Gao et al., 2022; Xiong et al., 2022a). Generally, the accumulation of large-granule starch is completed preferentially at the early stage of grain filling. With the advancement of grain filling, large-granule starch gradually decomposes into small-granule starch (Jiang et al., 2022a), which in turn leads to a decrease in the proportion of large-granule starch and an increase in the proportion of small-granule starch. The relative crystallinity and 1045/1022 cm^-1^ value of starch can reflect the stability and order of starch crystals (Sevenou et al., 2002; Vandeputte et al., 2003). In this study, the N_1_ and N_2_ treatments reduced the relative crystallinity and order of starch granules of superior grains in both varieties, and with an increase in the proportion of postponed N topdressing, the stability of the starch crystals decreased. Significantly, the stability of starch crystals of superior grains of both varieties was the lowest under the N_2_ treatment. This is because the relative crystallinity and order degree of starch are mainly affected by the chain length distribution of amylopectin and the starch granule size (Chen et al., 2023). At the same time, both N_1_ and N_2_ treatments significantly increased the proportion of A chains and small granule starch in superior grains and significantly reduced the proportion of the (B2+B3) chain in both varieties, with the largest proportion of A chains and small granule starch and the smallest proportion of the (B2+B3) chain under N_2_ treatment. A higher proportion of the A chain is not conducive to the formation of a good double helix structure, which leads to starch crystal structure defects. In contrast, a lower proportion of the (B2+B3) chain may reduce the number of double helix structures that can cross the entire crystalline lamellae, resulting in a weakened binding force to maintain the internal structure of starch granules, thereby reducing the stability of the starch crystal structure (Wang et al., 2010; Witt et al., 2012; Zhang et al., 2013; Zhu et al., 2023). In addition, the reduction in starch granule size leads to a decrease in relative crystallinity and degree of starch order (Zhang et al., 2013). Previous studies have shown that the application of N fertilizers increases the relative crystallinity of starch in inferior grains (Jiang et al., 2022a). The results of this study are consistent with Jiang’s conclusions. Moreover, this study further showed that the order of starch granules in inferior grains increased, and the disorder of starch granules decreased under N_1_ and N_2_ treatments. This indicates that the application of nitrogen fertilizer can improve the crystal structure of starch in inferior grains and the order of starch crystals, which further complements the results of Jiang et al. (2023) and Yang et al. (2020). However, this study also showed that increasing the proportion of postponed N topdressing significantly reduced the relative crystallinity and order of starch granules in inferior grains, which may be related to the high amylose content under the N_2_ treatment. Higher amylose content destroys the double-helix structure of the starch crystalline region, thus reducing the relative crystallinity of the starch (Jiang et al., 2023).

Starch gelatinization is an endothermic process. The essence of gelatinization is the loss of the starch double-helical structure, expressed as the gelatinization enthalpy (Zhu et al., 2023). Kong et al. (2015) showed that low amylose content and high relative crystallinity are associated with high gelatinization enthalpy. Similar results were observed in this study. In this study, the N_1_ treatment resulted in a low amylose content and high relative crystallinity of two varieties, while N_2_ had the opposite trend. This is an important reason why the N_1_ treatment increased the gelatinization enthalpy of the superior grains, whereas the N_2_ treatment reduced the gelatinization enthalpy. In addition, this study found that N application reduced the gelatinization enthalpy of inferior grains of two varieties, and the gelatinization enthalpy of inferior grains under the N_2_ treatment was the lowest. This is related to the increase in the proportion of the A chain under N_2_ treatment, leading to the instability of the double-helix structure formed in the crystalline region, which can be dissociated at a lower temperature. Thus, it has a lower gelatinization enthalpy (Li et al., 2020). Starch retrogradation is defined as the recrystallization of starch molecular chains that differ from the original structure (Martinez et al., 2018). Amylose content and amylopectin chain length distribution have been proven to be important factors affecting starch retrogradation (Li et al., 2020; Li et al., 2022). Many studies have shown that amylose has a synergistic effect on the retrogradation properties of starch (Li et al., 2022; Yang et al., 2022b; Jiang et al., 2023), and that amylose molecules crystallize rapidly during starch retrogradation, thereby increasing the enthalpy and degree of retrogradation (Jiang et al., 2023). In addition, the A and longer B1 chains in amylopectin enhance the mobility of molecules by forming intermolecular hydrogen bonds and increasing the starch retrogradation rate (Martinez et al., 2018). In this study, both N_1_ and N_2_ treatments reduced the retrogradation enthalpy of inferior grains of the two varieties, especially the N_1_ treatment further reduced the retrogradation degree and improved the retrogradation properties of inferior grain starch. This may be related to the low amylose content and the highest proportion of amylopectin B1 chains in the inferior grains under the N_1_ treatment (**Figure 1**, **Table 5**).

In summary, compared with N_1_, N_2_ treatment increased the proportion of small starch granules and the proportion of (A+B1) chains in the superior and inferior grains of the two varieties, and decreased the relative crystallinity and 1045/1022 cm^-1^ value, resulting in a decrease in starch crystal stability. Compared with superior grains, inferior grains had smaller starch granules, more uneven surface of starch granules, thereby the crystal structure of inferior grains was more unstable. Based on the reduction of nitrogen application (150 kg N hm^-2^) using a postponing N topdressing treatment helped to reduce the stability of starch crystal structure, improve starch thermal characteristics, and especially reduce the inferior grains gelatinization enthalpy. These results helped to achieve the improvement of the taste quality of the whole spike of grains through the enhancement of the taste value of inferior grains based on the stabilization of high yields.

### 4.2 Relationship between starch structure, thermal properties of superior and inferior grains and rice eating quality index

Starch structural properties are closely related to the cooking and eating quality characteristics. At present, there are several studies on the effects of starch structure and physicochemical properties on rice eating quality under N fertilizer management, but the conclusions are inconsistent (Xiong et al., 2022a; Yang et al., 2022b; Jiang et al., 2023). Xiong et al. (2022a) showed that the hardness of rice, amylose content, starch granules size, the proportion of amylopectin A chain and B1 chain showed a trend of decreasing first and then increasing as the proportion of postponing N topdressing, and the N application management of the base: tiller: panicle fertilizer=5:2:3 helped to increase the breakdown value, gelatinization enthalpy, reduce the setback value, improve the starch gelatinization properties, and ultimately reduce the hardness of rice and improve the taste. Yang et al. (2022a) reported that postponing N topdressing increased the proportion of amylopectin A and B1 chains, thereby increasing the breakdown value, peak viscosity, and taste value.

This study showed that varieties and nitrogen fertilizer had significant effects on the taste value of superior and inferior grains of the two varieties. Compared with the low-taste variety, the taste value of inferior grains of the high-taste variety significantly increased by 10.34-10.84%. This indicates that the difference in the taste value of inferior grains is an important reason for the overall taste value difference between the high- and low-taste varieties. In this study, N application treatments reduced the taste value of superior grains in both varieties, with the N2 treatment showing the smallest decrease in taste value. However, increased the taste value of inferior grains in both varieties under N application treatments. The taste value of inferior grains was the greatest under the N2 treatment, followed by N1 treatment and N0 treatments. Starch granule size affects the taste of the superior grains. The content of large starch granules and the average diameter of starch granules had a synergistic effect on the taste value of the superior granules (**Figure 6**). This may be because the average diameter of large and relatively large starch granules increases the surface area for binding with water molecules, which helps water molecules enter the interior of the starch granules to improve their swelling properties (Zhu et al., 2021). Studies have confirmed that the high relative crystallinity of large-granule starch is due to its rich B2 and B3 amylopectin chains (Takeda et al., 1999). This is also why the proportion of large-granule starch and the (B2+B3) chain was positively correlated with the taste value of superior grains in this study. In addition, this study also suggested that increasing the proportion of small granule starch, (A+B1) chains, would reduce the taste value of superior grains, which may be caused by the poor hydration properties of small granule starch rich in A and B1 chains (Takeda et al., 1999; Xiao et al., 2022).

In this study, the gelatinization and retrogradation enthalpies of superior grains were inversely proportional to the taste value of superior grains, which is inconsistent with the conclusion of Jiang et al. (2022a) that the taste value of superior grains is inversely proportional to the gelatinization enthalpy and positively proportional to the retrogradation enthalpy and degree of retrogradation. This may be related to the selection of rice varieties and differences in N fertilizer management. This study also found that increasing the amylopectin content would simultaneously increase the taste value of inferior grains. The small molecular weight of amylopectin components is easily leached from the swollen and broken starch granules during cooking, which increases the stickiness of rice and may be the reason for the increased taste value (Li et al., 2016; Zhang et al., 2023). In particular, the highest amylopectin content and stickiness of inferior grains under the postponed N topdressing treatment supported the higher taste value under postponed N topdressing. The effect of amylose content on the taste value of inferior grains is mainly reflected by an increase in amylose content, which simultaneously increases the hardness of rice and reduces its taste (Zhou et al., 2020). Significantly, in this study, the relative crystallinity and average diameter of the starch granules were important indicators for simultaneously improving the taste value of superior and inferior grains.

The hardness and stickiness of rice are important indicators of its textural properties. Rice hardness is significantly correlated with protein and amylose content (Xiong et al., 2022b). Previous studies have shown that N application treatments increase the protein content, hinder damage to the short-range ordered structure by the entry of water molecules into starch during cooking, and inhibit starch granule swelling and gelatinization, thereby increasing rice hardness (Shi et al., 2023). In addition to co-crystallization with long chains of amylopectin, amylose forms amylose-lipid complexes with lipids to inhibit starch leaching during cooking or the swelling of starch granules during heating, ultimately increasing the hardness of cooked rice (Li and Gilbert, 2018). Stickiness is caused by the exudation of small-molecule amylose and amylopectin, specifically short chains of amylopectin, onto the surface of rice grains during cooking (Li et al., 2016; Li and Gilbert, 2018). In addition, an increase in amylopectin short-chain (DP ≤ 36) content reduces rice hardness (Xiong et al., 2022b). However, a previous study showed that an increase in the proportion of amylopectin A and B1 chains simultaneously increases the hardness of rice, whereas an increase in the proportion of amylopectin B2 and B3 chains simultaneously increases the stickiness of rice (Xiong et al., 2022a).

In this study, the high-taste variety Shuangyou 573 had higher stickiness and low hardness of superior and inferior grains compared to the low-taste variety Fyou 498. The difference in amylose content was the main reason for the difference in rice texture between the two varieties (**Figure 1**). N application increased the hardness of the superior grains of both varieties and reduced their stickiness. This is because the N_1_ and N_2_ treatments increased the proportion of amylopectin (A+B1) chain and decreased the proportion of (B2+B3) chains in both varieties (**Table 2**), which is consistent with the results of Xiong et al. (2022a). However, this study also showed that the hardness and stickiness of superior grains in two varieties increased with an increase in the proportion of N topdressing. N_2_ treatment increases the amylose content, amylopectin content, and amylopectin (A+B1) chain ratio of superior grains; thus, the small-molecular-weight amylopectin and short-chain amylopectin of rice grains are preferentially leached during cooking, increasing the stickiness of rice (Li and Gilbert, 2018; Zhang et al., 2023). Subsequently, amylose components and long-chain amylopectin are leached; however, long-chain amylopectin can easily maintain the integrity of starch granules, which bond and co-crystallize with amylose, thereby increasing the heat resistance of the starch crystal structure, hindering starch granule swelling and starch solubility, and ultimately increasing the hardness of the rice grains (Ong and Blanshard, 1995; Yi et al., 2022). In addition, the crystal structure of starch, gelatinization enthalpy, and proportion of small granules in this study were significantly correlated with the hardness and stickiness of superior granule rice. Consistent with the trend of strong grains, N_2_ treatment of the two varieties of inferior grains hardness, stickiness maximum. This was related to the increase in amylose, amylopectin content, and short chain of amylopectin by N_2_ treatment, which further supplemented the results of Zhang et al. (2023). In addition, Jiang et al. (2023) reported that large granule starch which was rich in B2 and B3 chains has a more stable crystal structure and higher relative crystallinity. During gelatinization, the long side chains of amylopectin are entangled with amylose, which limits the expansion of starch granules and increases their hardness. This is more similar to our study, i.e., long-chain amylopectin, the proportion of large granular starch, and relative crystallinity were all significantly positively correlated with the hardness and negatively correlated with the viscosity of inferior grains. In conclusion, compared with N_1_, N_2_ treatment had a more significant effect on the taste value of superior and inferior grains, especially the inferior grains. It was shown that inferior grains of high-yielding and high-taste value varieties should be screened to synergistically enhance the taste value of superior and inferior grains by enhancing cooked rice stickiness and moderately increasing hardness based on reduction of nitrogen application (150 kg N hm^-2^) using a postponing N topdressing treatment (basal fertilizer: tillering fertilizer: panicle fertilizer=3:1:6), which provides theoretical information for high-quality rice production.

## 5 Conclusion

Inferior grains had lower (B2+B3) chains of amylopectin, smaller starch granules, the surface of starch granules was more uneven and more pitting. Compared with N_1_, N_2_ treatment increased the proportion of small granule starch, the ratio of (A+B1) chains, amylose, amylopectin, and total starch content in the superior and inferior grains of the two varieties, decreased the relative crystallinity and the value of 1045/1022 cm^-1^, which contributed to the decrease in the stability and order of the starch crystal structure, the decrease in the enthalpy of pasting, and finally increased the taste value, hardness, and stickiness of the two varieties of superior and inferior grains. Correlation analysis showed that the relative crystallinity and average diameter of the starch granules were significantly positively correlated with the taste value of the superior and inferior grains and can be used as an evaluation index for the simultaneous improvement of the taste value of the superior and inferior grains.

## Data availability statement

The original contributions presented in the study are included in the article/supplementary material. Further inquiries can be directed to the corresponding author.

## Author contributions

XY mapped and wrote the original draft. YL, YY, KC, and YW performed the experiments and collected the data. HYL and BL contributed to the methodology. YM contributed to the formal analysis. CG, ZC, ZY, and YS revised the manuscript. JM and YS administered the project and provided funding acquisition. All authors contributed to the article and approved the submitted version.
